# Coronary physiology assessment in a cardiac transplant patient

**DOI:** 10.1007/s12471-019-1300-z

**Published:** 2019-07-08

**Authors:** L. J. C. van Zandvoort, K. Masdjedi, M. N. Tovar Forero, O. Manintveld, J. Daemen

**Affiliations:** Department of Cardiology, Erasmus University Medical Center, Rotterdam, The Netherlands

**Fig. 1:**
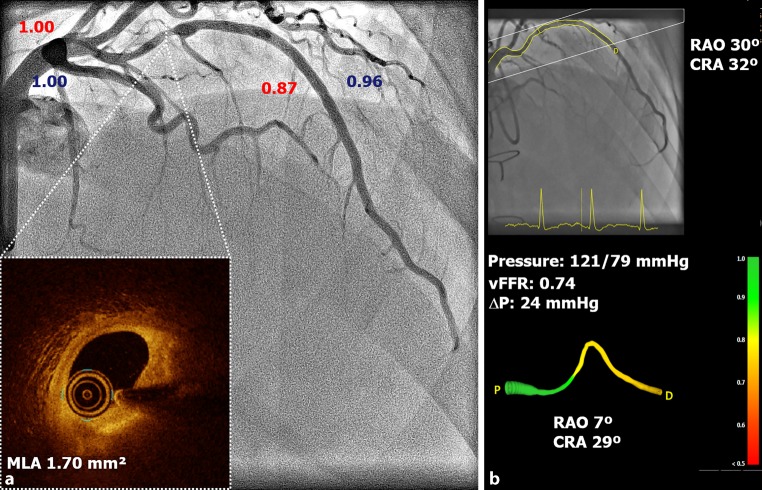
**a** Coronary angiography, 14 years after allograft cardiac transplant. The LAD shows an angiograpic intermediate stenosis in the midsegment; Pd/Pa values in blue and FFR values in red. Optical coherence tomography of the LAD shows a 15 mm lesion with a minimal lumen area (MLA) of 1.70mm^2^ and appropriate landing zones. **b** Vessel FFR (vFFR) of the LAD. The vFFR is 0.74, which indicates a significant lesion (threshold ≤0.80). *LAD* left anterior descending coronary artery, *Pd/Pa* distal coronary artery pressure/aortic pressure, *FFR* fractional flow reserve

A 39-year-old male underwent coronary angiography 14 years after cardiac allograft transplantation revealing an intermediate grade stenosis in the mid left anterior descending artery (LAD) for which further physiological assessment was performed (Fig. 1). Subsequent pressure wire-based fraction flow reserve (FFR_pw_) was 0.87, suggesting a hemodynamically non-significant lesion. However, non-hyperaemic 3‑dimensional quantitative coronary angiography-based vessel fractional flow reserve (vFFR) was 0.74 (Fig. 1b). Given the discrepancies, optical coherence tomography was performed showing a fibrofatty plaque with a minimal lumen area (MLA) of 1.70mm^2^. The LAD was subsequently treated with a 3.0 × 15 mm stent. There has been ongoing debate on the validity of using FFR in denervated hearts due to high rates of microvascular dysfunction and an unreliable hyperaemic response [1]. Angiography based vFFR might be a promising new technology to study the hemodynamic significance of intermediate coronary artery lesions in denervated hearts [2].
